# A Case of Brachial Plexus Schwannoma Intrathoracic Extension Guided via Video-Assisted Thoracoscopic Surgery (VATS)

**DOI:** 10.7759/cureus.77368

**Published:** 2025-01-13

**Authors:** Tuba Sahinoglu, Atilla Can, Halil Sen, Derya Ozer, Huseyin Yildiran

**Affiliations:** 1 Department of Thoracic Surgery, Selcuk University Faculty of Medicine, Konya, TUR; 2 Department of Otorhinolaryngology, Osmaniye State Hospital, Osmaniye, TUR

**Keywords:** brachial plexus, intrathoracic extension, pleural cavity, schwannoma, video-assisted thoracoscopic surgery

## Abstract

Schwannomas, arising from the brachial plexus, are rare nerve sheath tumors originating from Schwann cells, affecting peripheral nerves. Brachial plexus schwannomas present unique diagnostic and surgical challenges due to their rarity and intricate anatomy. This case report focuses on a significant case of a left brachial plexus schwannoma extending into the pleural cavity, emphasizing the complexities in diagnosis and the use of video-assisted thoracoscopic surgery (VATS) for intrathoracic extensions.

A 71-year-old female with a two-year history of left arm pain and numbness presented, having undergone cervical disc herniation surgery without improvement. Imaging revealed a 23 mm cystic lesion extending into the thorax. VATS, supported by a multidisciplinary team, successfully excised the intrathoracic schwannoma originating from the brachial plexus lower trunk. Postoperative recovery was uneventful, confirming grade 1 schwannoma on histopathology. However, the patient reported left arm numbness during follow-up, leading to electromyography revealing axonal neuropathy and plexopathy. Rehabilitation and medication were initiated.

Brachial plexus schwannomas, though benign, pose diagnostic challenges, often mimicking other conditions. Accurate diagnosis involves clinical evaluation, imaging, and sometimes biopsy. VATS, a less invasive alternative, proved effective in this case, shortening recovery time and improving patient satisfaction. The multidisciplinary approach, including chest surgery in intrathoracic cases, is crucial for optimal management. Surgical resection, while preserving nerves, is the primary treatment, although challenges may lead to postoperative complications.

Intrathoracic brachial plexus schwannomas demand a comprehensive approach where VATS significantly enhances diagnostic accuracy and patient outcomes.

## Introduction

Schwannomas are benign, encapsulated nerve sheath tumors that arise from Schwann cells situated along the nerves. These tumors can affect the third to twelfth cranial, peripheral, and autonomic nerves [1,2]. Among them, brachial plexus schwannomas are particularly rare, representing only 5% of all cases originating from the brachial plexus [3,4]. The challenge in diagnosing and surgically managing brachial plexus schwannomas stems not only from their rarity but also from the complex anatomy of the brachial plexus, which complicates both the identification and treatment of these lesions [2-5].

Brachial plexus schwannomas can present with a range of neurological symptoms depending on their size and location. When these tumors extend into the pleural cavity, they pose an additional challenge due to the proximity to vital structures and the difficulty of surgical access. In this context, the use of minimally invasive techniques, such as video-assisted thoracoscopic surgery (VATS), has proven advantageous. VATS allows for enhanced visualization and precision during surgery, leading to improved outcomes and reduced complication rates compared to traditional open procedures. The utilization of VATS in the management of brachial plexus schwannomas with intrathoracic extension represents an innovative and effective approach to such rare cases.

This case report presents a rare instance of a brachial plexus schwannoma with intrathoracic extension into the left pleural cavity, leading to numbness in the patient’s left arm. The case highlights both the diagnostic and surgical challenges inherent in managing such rare tumors. The surgery was performed using a multidisciplinary approach, with thoracic surgeons, otorhinolaryngologists, and other specialists collaborating to achieve optimal outcomes. Postoperatively, neurologists and the physical therapy department played a crucial role in the patient’s rehabilitation, focusing on the management of numbness and any residual symptoms. VATS was employed to address the thoracic extension of the tumor, demonstrating the feasibility and advantages of this minimally invasive approach.

This article was previously presented as a poster at the 2024 UASK (Ulusal Akciğer Sağlığı Kongresi, International Lung Health Congress) Annual Scientific Meeting on March 7, 2024.

## Case presentation

A 71-year-old female patient presented to our clinic with complaints of pain and numbness in her left arm for the past two years. It was revealed in her medical history that she had asthma and had undergone cervical disc herniation surgery a year ago due to her current complaints. According to the patient’s statement, there was no improvement in her symptoms after the surgery. Physical examination did not reveal any loss of muscle strength or sensation in the left upper extremity. Informed consent has been obtained from the patient.

**Figure 1:**
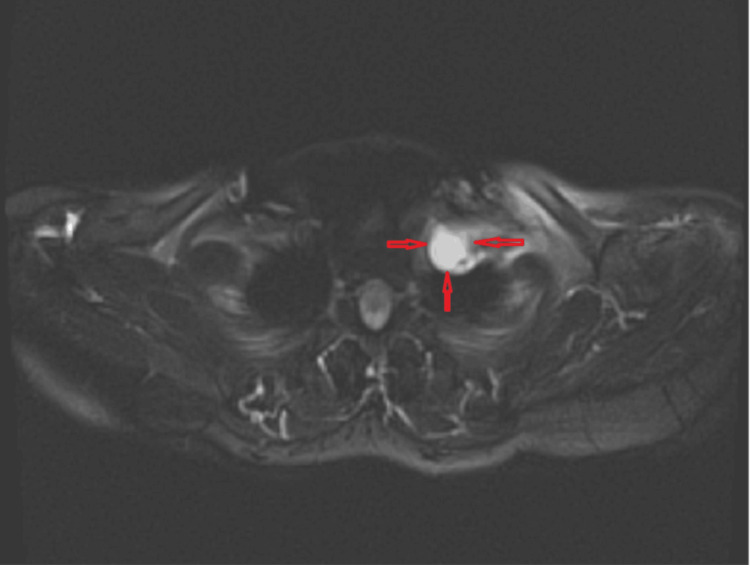
Axial section of magnetic resonance imaging showing the location and appearance of the lesion.

**Figure 2:**
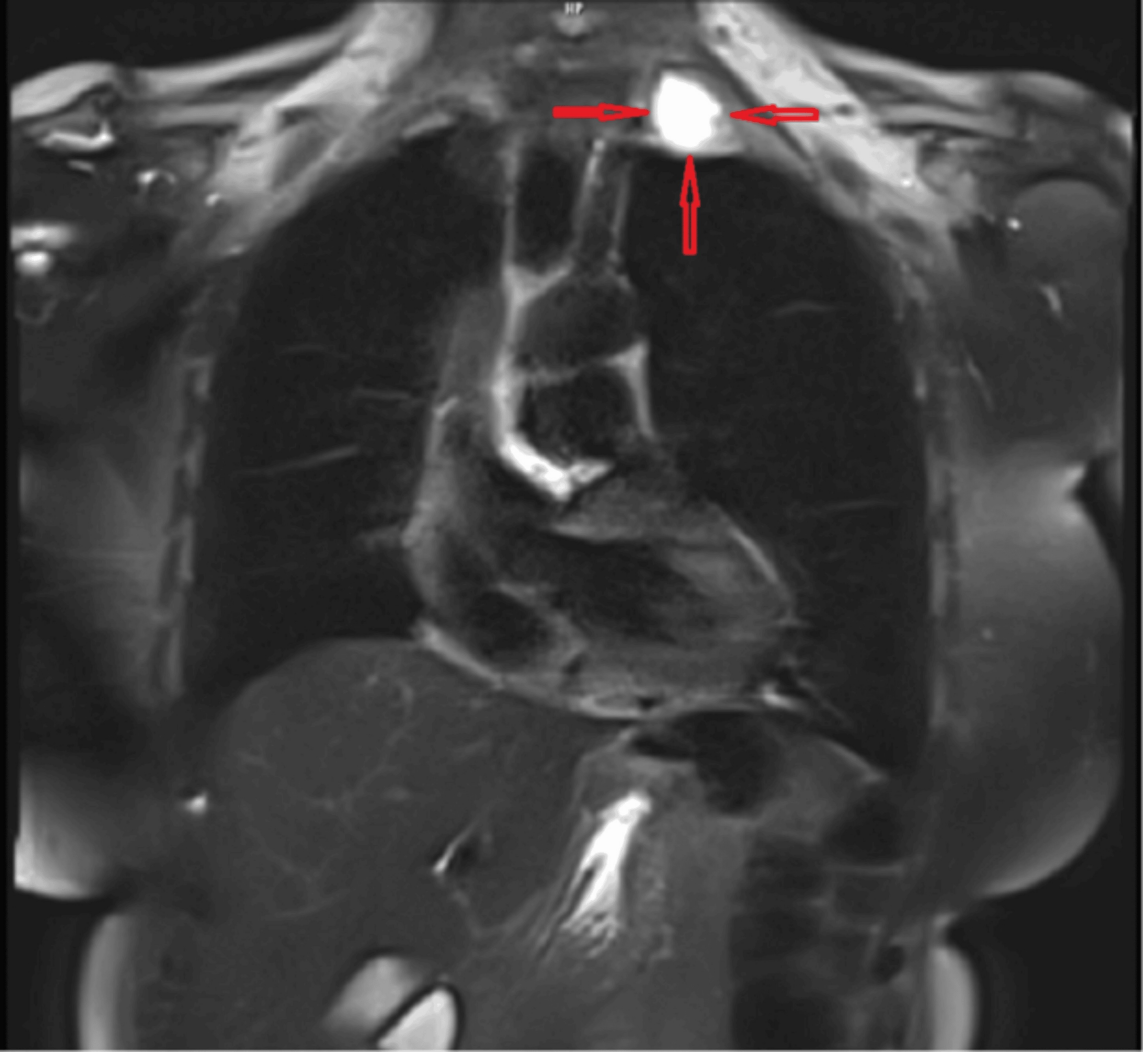
Coronal section of magnetic resonance imaging showing the location and appearance of the lesion.

**Figure 3:**
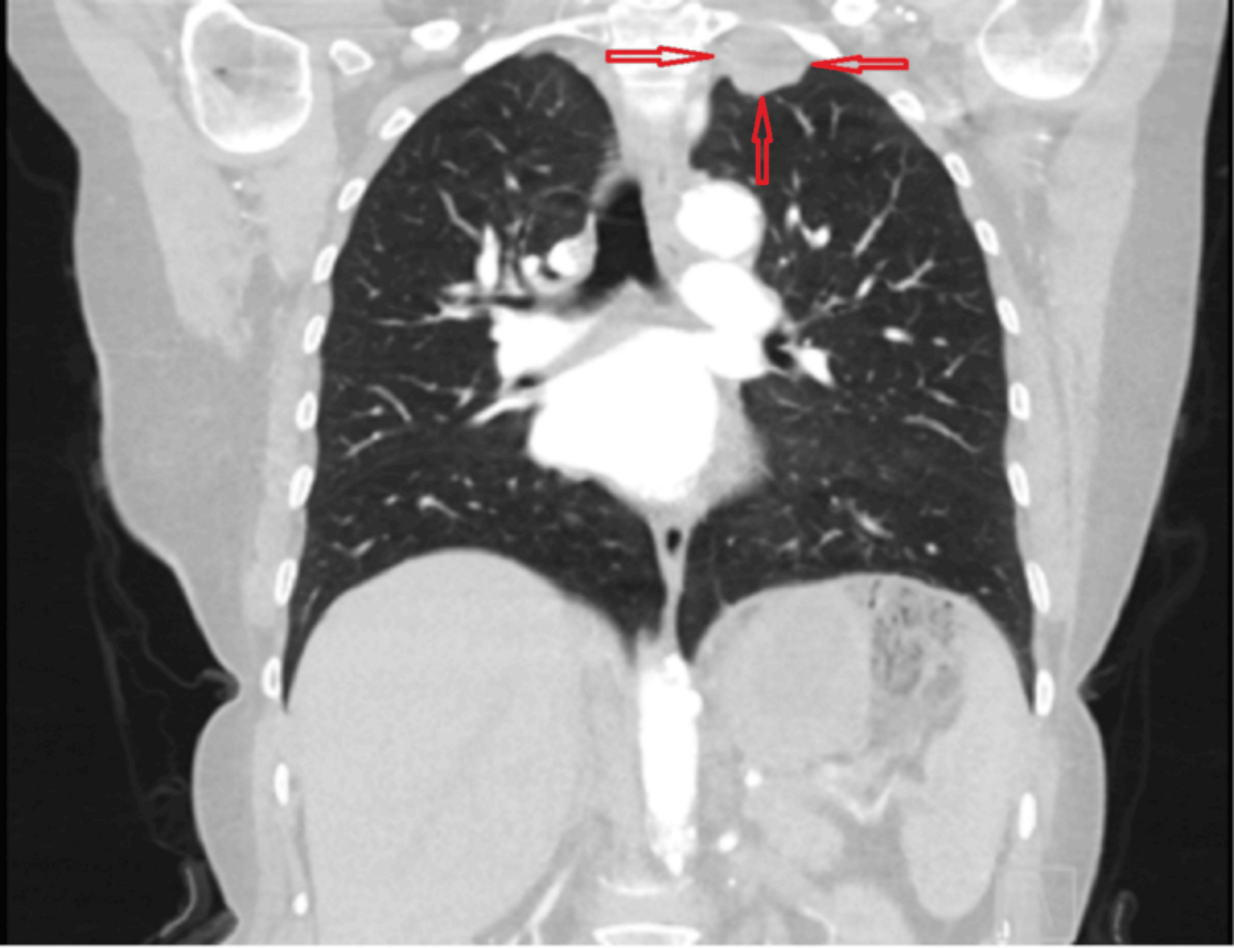
Coronal section of thoracic computed tomography showing the location and appearance of the lesion.

**Figure 4:**
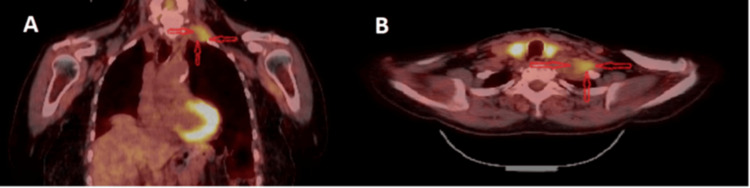
A) The PET (Positron emission tomography) coronal section shows the localization of the lesion and its corresponding FDG (Fluorodeoxyglucose) uptake, providing a clear view of the tumor’s metabolic activity and spatial relationship with surrounding structures. B) The PET axial section provides a detailed visualization of the lesion’s localization and FDG uptake, offering additional insight into the tumor’s characteristics and its interaction with nearby tissues.

The laboratory tests, including complete blood count (CBC), liver and kidney function tests (LFT, RFT), electrolyte levels, C-reactive protein (CRP), and blood glucose levels, were all within the normal range. The patient’s values were as follows: alanine aminotransferase (ALT) 11 U/L (reference: 0-33 U/L), aspartate aminotransferase (AST) 26 U/L (reference: 0-35 U/L), creatinine 0.61 mg/dL (reference: 0.50-0.90 mg/dL), urea 29 mg/dL (reference: 16.6-48.5 mg/dL), glucose 100 mg/dL (reference: 72-106 mg/dL), sodium (Na) 140 mmol/L (reference: 136-145 mmol/L), potassium (K) 4.18 mmol/L (reference: 3.5-5.1 mmol/L), hemoglobin (Hb) 12.5 g/dL (reference: 12-15.5 g/dL), white blood cells (WBC) 4.74 × 10³/µL (reference: 3.5-10.5 × 10³/µL), and CRP <3.64 mg/L (reference: 0-5 mg/L), with no significant abnormalities noted. A left apical lesion was detected on a chest X-ray, and a well-defined 23 mm soft tissue density lesion adjacent to the left subclavian artery, extending into the thorax, was observed on chest computed tomography (CT) (Figure 1). Magnetic resonance imaging (MRI) and positron emission tomography (PET) were planned for a detailed examination. The lesion, evaluated as cystic, was located in the apical segment of the left upper lobe on lung-mediastinum MRI (Figures 2-3). PET imaging showed fluorodeoxyglucose F18 (FDG) uptake (SUVmax: 5.00), a positron-emitting radiotracer used with PET to diagnose and monitor various conditions (Figure 4). Standard imaging modalities such as X-ray, CT, and MRI provide detailed visualization of healthy and diseased tissues.

**Figure 5:**
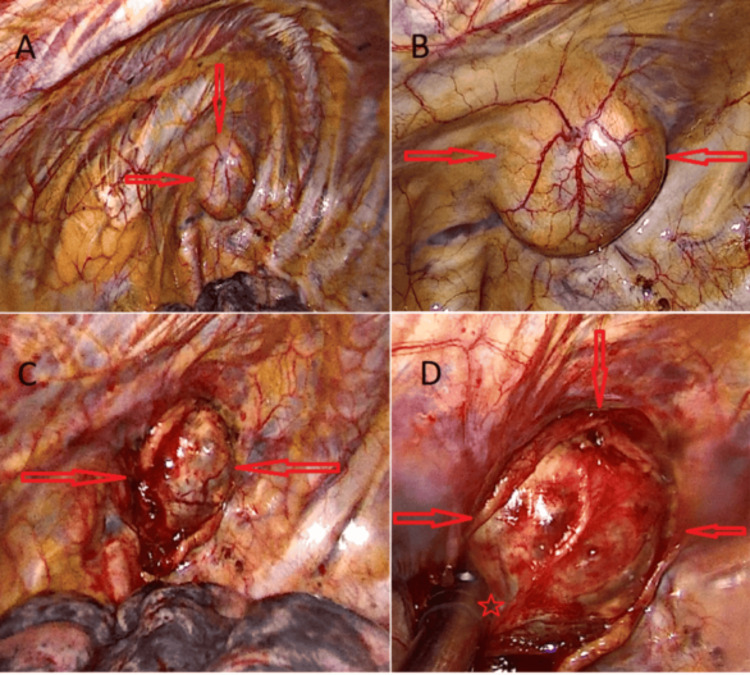
A) Location and appearance of the lesion during video-assisted thoracoscopic surgery (VATS) exploration. B) Capsule and vascular structure of the lesion during VATS exploration. C) VATS image of the lesion after the parietal pleura has been dissected. D) VATS image showing the continuation of the lesion’s nerve root and cervical extension, with the nerve root marked by an asterisk.

The patient was fully informed about her condition, and after discussing the available treatment options, VATS was planned as the most appropriate approach. Under general anesthesia, a double-lumen endotracheal tube was placed, and the thoracic surgery was performed in the lateral decubitus position with two ports, without the need for a utility incision. During VATS exploration, a cystic lesion located in the cupula with intrathoracic extension was observed (Figure 5).

The intrathoracic portion was thoroughly dissected using sharp and blunt dissection techniques. As the lesion extended toward the supraclavicular area, the otorhinolaryngology team was consulted, and the patient was repositioned to the supine position. An approximately 7-8 cm cervical vertical J-incision was performed in the left supraclavicular region. Exploration revealed that the cystic lesion originated from the brachial plexus lower trunk. The lesion was carefully dissected from surrounding tissues, and the cystic content was aspirated. Nerve branches extending to the originating brachial plexus were preserved, and the remaining cyst wall was excised. The intactness of the phrenic nerve, brachial plexus, and branches of the lower trunk from which the mass originated were confirmed using a neurostimulator. Specimens were sent for histopathological examination. Postoperatively, pain control was effectively managed with paracetamol alone.

The patient was discharged on the 4th day of postoperative follow-up without additional complications. The pathology report indicated a grade 1 schwannoma with diffuse strong immunopositivity for S-100.

During outpatient clinic visits, the patient complained of numbness in the left upper extremity, prompting the request for electromyography (EMG). The EMG results revealed complete axonal neuropathy of the left accessory nerve and incomplete moderately severe axonal plexopathy of the lower trunk in an axonal pattern. Based on these findings, the Physical Medicine and Rehabilitation specialist recommended physiotherapy, and oral duloxetine 60 mg once daily was prescribed. Duloxetine is a serotonin-norepinephrine reuptake inhibitor (SNRI) that helps in managing pain and improving overall function by increasing the levels of serotonin and norepinephrine in the brain.

## Discussion

Brachial plexus schwannomas are rare nerve sheath tumors arising from Schwann cells. They are responsible for insulating peripheral nerves and can occur anywhere along the brachial plexus, a complex network of nerves that controls the movement and sensation of the upper extremities. While benign, brachial plexus schwannomas can present significant challenges in diagnosis and treatment.

Brachial plexus schwannomas often manifest as slow-growing masses, leading to a delayed diagnosis. Patients commonly present with symptoms such as pain, numbness, weakness, or a palpable mass in the shoulder or upper arm [6]. Since the lesion extended toward the thorax in our patient, there was no palpable mass in the neck region. However, in line with the literature [1,3], the patient exhibited pronounced pain and numbness in the left arm. The slow progression of symptoms can mimic other conditions, making accurate diagnosis a challenging task. The numbness and pain complaints in the arm were associated with the patient’s existing cervical disc, and surgery was performed; however, our patient did not experience any benefit. Additionally, the rarity of these tumors necessitates a high index of suspicion among clinicians [6].

Accurate diagnosis of brachial plexus schwannomas involves a combination of clinical evaluation, imaging studies, and, in some cases, biopsy. MRI is the preferred imaging modality, providing detailed information about the tumor’s location, size, and relationship to surrounding structures. EMG and nerve conduction studies may aid in assessing the extent of nerve involvement. Biopsy, though often reserved for atypical cases, can confirm the Schwann cell origin of the tumor [6,7].

VATS is a less invasive approach compared to traditional surgery. This can potentially shorten the patient’s postoperative recovery time and reduce the duration of hospitalization. Moreover, the association of VATS with less pain, smaller incisions, and faster recovery may enhance patient satisfaction [8]. In this case, with intrathoracic extension, the VATS approach facilitated the release of the tumor, improving visibility in the cervical region. Two port incisions were made in the patient’s thoracic area. The chest tube was removed on the 2nd postoperative day, and the patient was discharged on the 4th day. Managing brachial plexus schwannomas requires a multidisciplinary approach involving neurosurgeons, orthopedic surgeons, oncologists, and rehabilitation specialists. Chest surgery is also included in the treatment of lesions growing intrathoracically like ours. Treatment options vary based on the tumor’s size, location, and degree of nerve involvement. Surgical resection is the primary treatment, aiming to achieve complete tumor removal while preserving nerve function.

Brachial plexus schwannomas are complex entities that pose significant surgical challenges due to the intricate anatomical nature of the brachial plexus. In some cases, complete resection of the tumor may not be feasible without risking significant nerve damage and neurological deficits [9]. As highlighted by Roh, intracapsular enucleation serves as a function-preserving alternative, which can effectively minimize nerve damage while addressing the tumor. This method allows for safe resection of the schwannoma while preserving nerve function in most cases, with minimal neurological complications postoperatively [4]. Additionally, the use of intraoperative neuromonitoring during surgical resection can provide a safer procedure by continuously assessing nerve function, thereby minimizing the risk of postoperative complications.

However, in cases where resection poses a high risk to the nerve or when the tumor is unresectable, a conservative approach may be considered. Regular monitoring, symptomatic management, and potential use of radiation therapy could provide effective alternatives. According to a study by Apicella et al., radiation therapy has shown efficacy in managing vestibular schwannomas, and while this is typically associated with a different tumor type, its principles, and outcomes could be considered for brachial plexus schwannomas, especially in cases of residual or unresectable tumors [10]. Radiation therapy may be a valuable adjunct in managing these challenging cases, providing a viable option when complete surgical resection is not possible.

Postoperative rehabilitation is an integral part of the recovery process, ensuring optimal functional outcomes. Physical therapy plays a crucial role in enhancing motor recovery and mobility after surgery or radiation therapy [11]. In our case, despite the successful excision of the lesion, the patient experienced some numbness in the left arm, likely due to surgical trauma. This was managed with further intervention from the physical therapy department, which played a key role in the patient’s recovery process.

## Conclusions

Brachial plexus schwannomas represent a rare and complex clinical entity that requires a comprehensive and individualized approach to management. Tumors of the brachial plexus pose a significant challenge due to the neuroanatomical complexity of the plexus and the limited literature available on managing such rare tumors. Schwannomas, which arise from the nerve sheath, are one such tumor type. These lesions are surgically curable through gross total resection, offering favorable outcomes, acceptable risks, and a low incidence of tumor recurrence. When the tumor demonstrates intrathoracic extension, the VATS approach can be planned as an intrathoracic extrapleural mass. Collaborating with medical teams from different specialties is essential for accurate diagnosis, effective treatment planning, and optimal patient outcomes. As research in neuro-oncology advances, ongoing efforts to refine diagnostic techniques and therapeutic strategies will contribute to improved outcomes for individuals affected by brachial plexus schwannomas.

## References

[1] Yasumatsu R, Nakashima T, Miyazaki R, Segawa Y, Komune S (2013). Diagnosis and management of extracranial head and neck schwannomas: A review of 27 cases. Int J Otolaryngol.

[2] Wang L, Ge L, Ren Y (2023). Case report: Combined cervical incision with an intercostal uniportal video-assisted thoracoscopic surgery approach for mediastinal brachial plexus schwannoma. Front Oncol.

[3] Jia X, Yang J, Chen L, Yu C, Kondo T (2016). Primary brachial plexus tumors: Clinical experiences of 143 cases. Clin Neurol Neurosurg.

[4] Roh JL (2022). Function-preserving intracapsular enucleation of brachial plexus schwannomas: Is it safe and effective?. Oral Oncol.

[5] Femia F, Junemann C, Ruffini E, Guerrera F (2022). Intraoperative neuromonitoring in thoracoscopic excision of brachial plexus schwannoma. Interact Cardiovasc Thorac Surg.

[6] Hems T, Parafioriti A, Thomas BP, Di Bernardo A (2024). An algorithmic approach to the management of peripheral nerve tumours. J Hand Surg Eur Vol.

[7] Kwee RM, Borghans RA, Bruls RJ, Fasen BA, Kuburic D (2022). Diagnostic performance of diffusion-weighted MR neurography as an adjunct to conventional MRI for the assessment of brachial plexus pathology. Eur Radiol.

[8] Lyberis P, Balsamo L, Fontana EC, Ruffini E, Nicosia S, Roffinella M (2023). VATS phrenic nerve harvesting for brachial plexus neurotization: Literature review and our experience. Minerva Surg.

[9] Zhu XS, Song N, Song NC (2018). Comparison of the perioperative outcomes in antero-superior mediastinal tumor resection performed by transcervical resection and video-assisted thoracoscopic surgery. J Thorac Dis.

[10] Apicella G, Paolini M, Deantonio L, Masini L, Krengli M (2016). Radiotherapy for vestibular schwannoma: Review of recent literature results. Rep Pract Oncol Radiother.

[11] Lubelski D, Pennington Z, Ochuba A, Azad TD, Mansouri A, Blakeley J, Belzberg AJ (2022). Natural history of brachial plexus, peripheral nerve, and spinal schwannomas. Neurosurgery.

